# Two-Y Crushing Technique: A Simple Technique to Crack the Nucleus in a Posterior Polar Cataract Using Two-Y Rotators

**DOI:** 10.7759/cureus.63416

**Published:** 2024-06-28

**Authors:** Balamurugan Ramatchandirane, Divya Sindhuja Pathuri, Mansi D Devalla, Shubhangi SN Prasad, Premika Pandarasamy

**Affiliations:** 1 Ophthalmology, All India Institute of Medical Sciences, Mangalagiri, Mangalagiri, IND

**Keywords:** two-y crushing technique, nucleofractis, manual phacoemulsification, sculpting nucleus, cracking nucleus, phacoemulsification, posterior polar cataract

## Abstract

Cataract surgeries in posterior polar cataracts (PPCs) are always challenging for an ophthalmologist, in spite of multiple techniques described for phacoemulsification surgery. The most important objective of cataract surgeries in PPCs is to achieve maximum removal or debulking of the nucleus so that, if any complications happen, they can be easily manageable. We describe a new technique to manually crush the nucleus after it is manually prolapsed out of the bag into multiple pieces with the help of two Y-rotators which were then removed by using a phacoemulsification probe so that complications of posterior capsular rupture because of inadvertent rotation of the nucleus during the phacoemulsification part could be avoided.

## Introduction

A posterior polar cataract (PPC) is a type of congenital cataract that may remain stationary or progressive in nature [1]. The main concern for cataract surgeries in PPCs is the intraoperative risk of posterior capsular rupture and associated complications like vitreous loss, etc., because of the pre-existing weakness or dehiscence of the posterior capsule [2]. Hence, the type of phacoemulsification technique has to be meticulously planned based on the grade of cataract, and maximal debulking or removal of the nucleus matter is most important before removal of the protective epinucleus sheet. In this article, we describe a new technique of cracking the nucleus for its removal by prolapsing it out of the bag and manually crushing it by using two "Y-rotators."

## Case presentation

**Figure 1:**
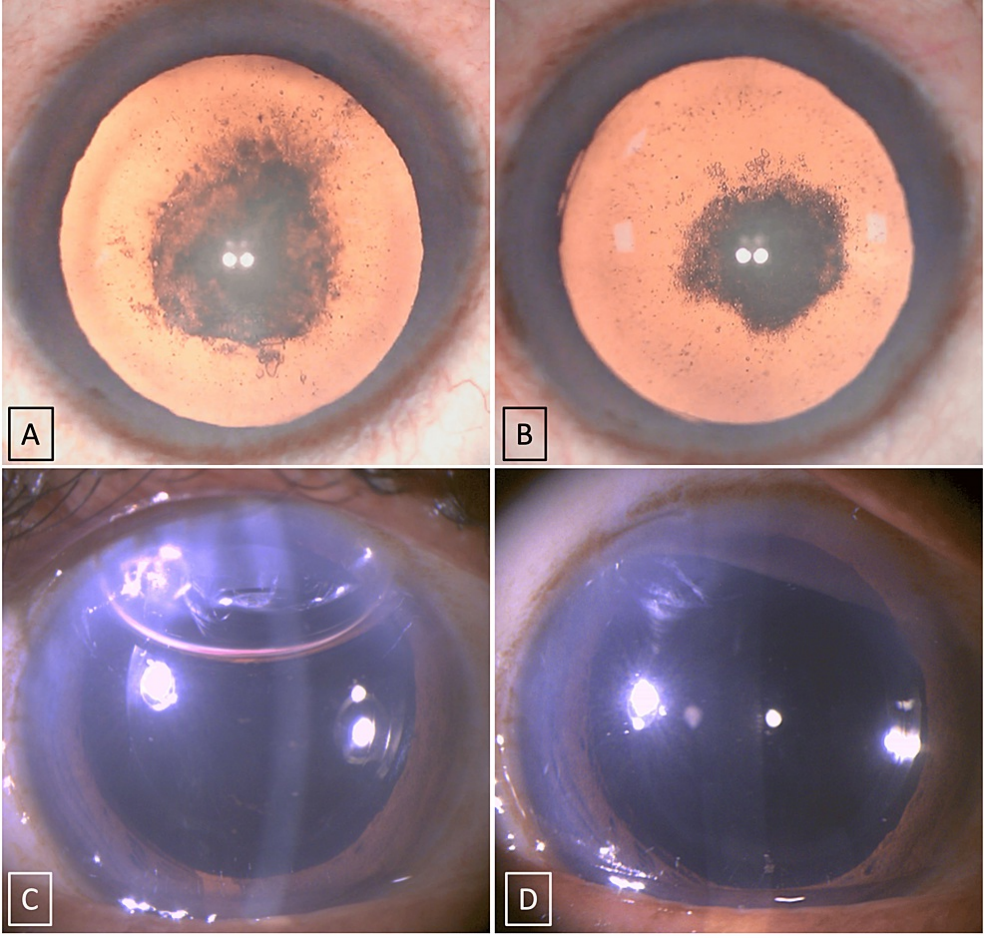
A and B show the preoperative images of the posterior polar cataracts of the right and left eye, respectively. C and D show the postoperative day 1 of the right eye and left eye, respectively, showing clear corneas and well-placed IOLs in the bag. IOL: Intraocular lens

**Figure 2:**
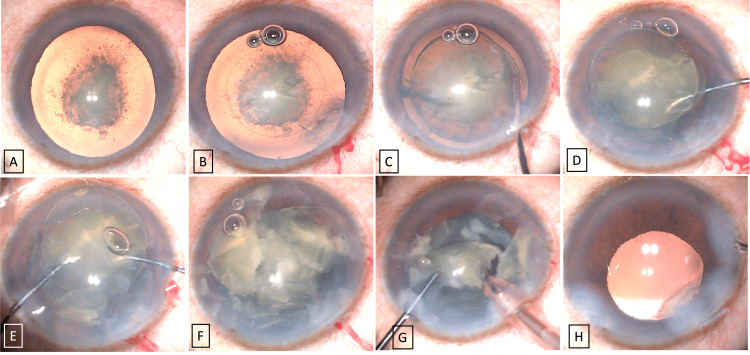
A shows the posterior polar cataract with dense posterior subcapsular plaque and hard nuclear sclerosis. B shows the round capsulorhexis of approximately 5 mm. C shows successful hydrodelineation with a well-cleaved nucleus from the epinucleus. D shows the prolapse of the nucleus using the Y rotator. E shows the manual crushing of the nucleus using two Y rotators. F shows the multiple pieces of the nucleus. G shows the emulsification of the nucleus using the phacoprobe. H shows the implantation of the single-piece IOL in the capsular bag. IOL: Intraocular lens

A male in his fifties presented with decreased vision in both eyes, which was more common during the last five years. On examination, his best corrected visual acuity was 6/36 (-4 DS) and 6/45 (-2 DS) for right and left eyes, respectively. Intraocular pressures of both eyes were normal. Under slit lamp examination, he had PPC with posterior subcapsular plaque, Grade I as per Daljit Singh classification [2], and nuclear sclerosis Grade III as per Emery-Little classification [3] in both eyes (Figures 1A, 2A, 1B). Anterior segment optical coherence tomography (ASOCT) has shown a thick plaque of PPC adherent to the posterior capsule, but there was no evidence of a pre-existing posterior capsular defect. His fundus examination was normal. Phacoemulsification cataract surgery using our new technique (the two-Y crushing technique) was done with the implantation of the posterior chamber intraocular lens (IOL) for the right eye followed by the left eye. Under topical anesthesia (Video 1), a clear corneal side port incision was made at 2 o'clock and 9 o'clock hour positions, and the main port incision was made at 11 o'clock hour positions. A viscoelastic material (sodium hyaluronate, 1.4%) was injected into the anterior chamber, and continuous curvilinear capsulorhexis (approximately 5 mm) was made (Figure 2B). Hydrodissection was avoided and hydrodelineation was attempted successfully to delineate the epinucleus from the nucleus (Figure 2C). The Y rotator (titanium made, Y-shaped blunt tip angulated at 45° and 0.7 mm width) was inserted through the 9 o' clock side port, inserted into the well-separated hydrodelineated ring, and gently pushed the nucleus upwards, which prolapsed the nucleus into the anterior chamber (Figures 2D). Now one more Y rotator was inserted through the 2’O clock side port, and using two Y rotators, the nucleus was crushed by pushing the two Y rotators close to each other and successfully cracking the nucleus into two pieces (Figure 2E). Similarly, the process was continued until four nucleus pieces were cracked (Figure 2F). Now, using viscoelastics (HPMC2%), the nucleus was pushed a little down in the iris plane and phacoemulsification was completed (Figure 2G). The air bubble between the nucleus and the cornea showed that the nucleus was not in touch with the endothelium. The epinucleus, along with the cortical matter, was later separated from the capsular bag using HMPC 2% (viscodissection) [4] and removed using an I and A (Irrigation and Aspiration) cannula. Finally, a single piece of hydrophobic foldable IOL was implanted and the viscoelastic was removed. All the corneal ports were sealed using hydration (Figure 2H). Postoperatively on day 1, both the eyes showed clear cornea with well-centered IOL and achieved BCVA of 6/6 with normal intraocular pressure (Figures 1C, 1D).

**Video 1:** Two-Y crushing technique in phacoemulsification in the posterior polar cataract.

## Discussion

Phacoemulsification of the nucleus in the PPC is a challenging technique, as the nucleus should not be rotated [4], and inadvertent rotation of the nucleus leads to the posterior capsular tear. Different kinds of techniques have been described such as lambda [5], trident [6], sideways sculpt technique [1], etc., all of which involve phacoemulsification in the bag technique, and attempting quadrant removal sometimes leads to inadvertent rotation of the nucleus. Our technique involves the nucleus completely prolapsing out of its bag and manually broken into multiple pieces using two Y rotators and then emulsifying it seems like a comfortable and safe technique as there is no rotation of the nucleus and less time is consumed, and the chamber is also well stable during the phacoemulsification. Kansas et al. [7] have described a manual phacofragmentation technique in which the phacofragmentation of the nucleus is done using a spatula and wire Vectis by pressing against each other in SICS (small incision cataract surgery). Kosakaran manually divided the nucleus into three pieces using the double nylon loop technique in SICS and extracted it through a 4.5 mm corneal incision [8]. To the best of our knowledge, this is the first time, phacoemulsification cataract surgery has been performed in PPC in which the manual prolapsing of the nucleus and cracking it using a two-Y rotator, like crushing an object with our hands, were done. We have also noticed that less cumulative dissipated energy was spent in this technique during quadrant removal, i.e., 1.80 for the right eye and 1.66 for the left eye, because no ultrasonic energy was used for phacofragmentation. The advantages of this technique are its simplicity and being easy to perform; no rotation of the nucleus occurs; less ultrasonic phacoenergy will be spent; debulking of the nucleus is easily carried out without touching the epinucleus sheet; and hence, posterior capsular rupture could be avoided during the phacoemulsification of the nucleus to some extent. This technique could be tried only when there is a well-clear-cut delineated demarcation ring noted between the nucleus and epinucleus during the hydrodelineation step (successfully hydrodelineation).

## Conclusions

There are several techniques described for sculpting and emulsification of the nucleus in the case of PPC and adoption of the technique in its entirety depends upon the surgeon's choice based on the condition and grade of the cataract. Phacoemulsification of the nucleus in cases of soft cataracts is easier, but when it comes to moderate or hard-grade cataracts, surgery is quite challenging as it carries the risk of rotating the nucleus and, in turn, the rupture of the PPC. Our technique of prolapsing and crushing the nucleus manually using two Y rotators into multiple pieces is simple and could be considered for moderate-hard grade nuclear sclerosis in PPCs. 
